# Immunotherapy Improves Clinical Outcome in Kirsten Rat Sarcoma Virus-Mutated Patients with Unresectable Non-Small Cell Lung Cancer Stage III: A Subcohort Analysis of the Austrian Radio-Oncological Lung Cancer Study Association Registry (ALLSTAR)

**DOI:** 10.3390/jcm14030945

**Published:** 2025-02-01

**Authors:** Elvis Ruznic, Marisa Klebermass, Barbara Zellinger, Brigitte Langer, Brane Grambozov, Ayurzana Purevdorj, Josef Karner, Georg Gruber, Markus Stana, Danijela Minasch, Karoline Kirchhammer, Claudia Steffal, Heidi Stranzl, Raphaela Moosbrugger, Petra Feurstein, Karin Dieckmann, Franz Zehentmayr

**Affiliations:** 1 Department of Radiation Oncology, Paracelsus Medical University, 5020 Salzburg, Austria; e.ruznic@salk.at (E.R.); b.zellinger@salk.at (B.Z.); b.grambozov@salk.at (B.G.); j.karner@salk.at (J.K.); m.stana@salk.at (M.S.); 2 Klinikum Ottakring, 1090 Vienna, Austria; marisa.klebermass@gesundheitsverbund.at (M.K.); brigitte.langer@gesundheitsverbund.at (B.L.); doktor@petra-feurstein.at (P.F.); 3 Klinikum Hietzing-Rosenhügel, 1090 Vienna, Austria; ayurzana.purvdorj@gesundheitsverbund.at; 4 Ordensklinikum Linz, 4020 Linz, Austria; georg.gruber@ordensklinikum.at; 5 Department of Radiation Oncology, Comprehensive Cancer Centre, Medical University Innsbruck, 6020 Innsbruck, Austria; danijela.minasch@tirol-kliniken.at; 6 Klinikum Klagenfurt, 9020 Klagenfurt, Austria; karoline.kirchhammer@kabeg.at; 7 Klinikum Favoriten, 1100 Vienna, Austria; claudia.steffal@gesundheitsverbund.at; 8 Department of Radiation Oncology, Comprehensive Cancer Centre, Medical University Graz, 8036 Graz, Austria; heidi.stranzl@medunigraz.at; 9 Department of Pulmonology, Paracelsus Medical University, 1090 Salzburg, Austria; r.moosbrugger@salk.at; 10 Department of Radiation Oncology, Comprehensive Cancer Centre, Medical University Vienna, 1090 Vienna, Austria; karin.dieckmann@akhwien.at

**Keywords:** durvalumab, KRAS mutation, KRAS G12C, chemoradiotherapy, radiation dose escalation

## Abstract

**Background/Objectives**: Current evidence suggests that patients with unresectable non-small cell lung cancer (NSCLC) whose tumours harbour driver mutations do not benefit from immune checkpoint inhibition. Kirsten rat sarcoma virus mutations (KRASmts), however, seem to be the exceptions to the rule. To this end, we compared KRASmt patients who were treated with immunotherapy to those without. **Methods**: ALLSTAR is a nationwide registry for patients with histologically verified non-operable NSCLC aged 18 or older having a curative treatment option. This report presents a subcohort of KRASmt patients who were recruited between 2020/03 and 2023/04. The diagnostic work-up included ^18^F-FDG-PET-CT scan and contrast-enhanced cranial CT or—preferably—MRI. Patients were treated with chemoradiotherapy (CRT) either followed by immune checkpoint inhibition (ICI) or not. **Results**: Thirty-two KRASmt patients with a median follow-up of 25.9 months were included in this analysis. After CRT, 27/32 (84%) patients received ICI. The 2-year overall survival rate in KRASmt patients who received immunotherapy was significantly better compared to those without ICI (N = 32; 84% versus 20%; *p* < 0.001). Likewise, the 2-year progression-free-survival with immunotherapy was also significantly better than in those without ICI (N = 32; 75% versus 20%; *p* < 0.001). Of the 12/32 patients (38%) who had received radiation doses > 66 Gy, none had a locoregional relapse, whereas in the other 20 patients, 5 (25%) events occurred (*p*-value = 0.116). **Conclusions**: Since KRASmt patients could benefit from ICI treatment, immunotherapy should be offered to these patients, similar to those without actionable genetic drivers. Additionally, radiation dose escalation > 66 Gy may also improve locoregional control in this subset of patients.

## 1. Introduction

Despite advances in all related disciplines, lung cancer is still the most common cause of cancer deaths worldwide [1]. The proportion of non-small cell lung cancer (NSCLC) amounts to approximately 80%, with 30% of the patients already presenting in locally advanced (LA) stages, i.e., UICC IIIa to IIIc [1].

Before the introduction of immune checkpoint inhibition (ICI) to clinical routine, the best possible median overall survival (mOS) achievable with chemoradiotherapy (CRT) alone was about 29 months [2], and the 5-year OS rates ranged between 15 and 30% [3]. With the approval of Durvalumab, the 5-year OS rates could be extended to 43% for patients receiving the PACIFIC regimen [4,5] with enduring disease control after five years [6]. Despite these encouraging results, 25% of the patients who were treated with immunotherapy progress within 18 months after Durvalumab consolidation therapy [7]. Locally advanced non-small cell lung cancer (LA-NSCLC) consists of a multitude of sub-entities, which results in a variety of treatment approaches, especially for those patients harbouring driver mutations. In this context, causes of early progression are poorly understood, and the benefit of ICI in these patients is doubtful [7,8]. Among these, Kirsten rat sarcoma virus mutations (KRASmts) seem to be the exception to the rule, in so far as these patients apparently benefit from ICI treatment. The reasons for this are not fully understood [8]. In a Western population, any KRAS mutation may occur in about 25% to 40% [8,9] of cases, with KRAS G12C being the most common oncogenic driver in general [8].

According to the notion that KRASmt patients might benefit from immunotherapy [8], the aim of the current project was to assess outcomes after chemoradioimmunotherapy (CRIT) in this subset of patients precisely. Since the KRASmt status was not reported in PACIFIC, our investigation contributes to a clarification of the limited understanding of immunotherapy response in this subset of patients.

## 2. Methods

### 2.1. Patients

Data on the current cohort were collected within the Austrian radio-oncological lung cancer study association registry (ALLSTAR) [10], which is a nationwide registry for unresectable NSCLC stage III patients. None of the patients participated in an early access programme (EAP), which marks a difference to other RWD studies [11,12,13]. At each centre, the patients were referred for CR(I)T after consensual decision in the multidisciplinary tumour board for thoracic malignancies. The local investigator, who had to be a board-certified radiation oncologist, entered the data in the web-based data capture (WBDC) system.

After approval by the lead ethics committee of the federal state of Salzburg on the 20th of March 2020 (approval number: Ethikkommission Land Salzburg Nr. 1002/2019), patients aged 18 or older were included in the registry if they had pathologically confirmed unresectable NSCLC UICC stage III according to TNM version 8. Patients without a curative treatment option were excluded. Detailed selection criteria were described elsewhere [10]. Twelve of the fourteen (86%) Austrian radiation oncology centres contributed to the whole dataset, from which only KRASmt patients were eligible for the current analysis. In contrast to PACIFIC [4,5], this registry also comprised patients with ECOG > 1. All patients provided written informed consent.

Initial diagnosis was based on contrast-enhanced whole body CT scan or—preferably—^18^F-FDG-PET-CT together with cranial MRI. As for histological or cytological verification, bronchoscopy or transthoracic needle aspiration with endobronchial ultrasound for mediastinal lymph node staging had to be performed. Finally, the baseline check-up also included pulmonary function tests (PFTs). Follow-up visits including contrast-enhanced thoracic CT and PFTs took place three months after the end of RT and on a biannual basis thereafter.

### 2.2. Radiochemoimmunotherapy

Radiotherapy (RT) was performed according to local practices at each of the participating centres with modern radiation techniques, such as 3D-RT or intensity modulated radiotherapy (IMRT), either in a step-and-shoot mode or through volumetric arc therapy (VMAT). Total radiation doses of 60–66 Gy in 2 Gy fractions were regarded as the standard of care (SoC), but altered fractionation schemes and dose escalation beyond 66 Gy were also possible. Since treatment regimens differed substantially between institutions, total radiation doses were compared with each other on the basis of biologically equivalent doses in 2 Gy fractions (EQD2) with *D* for total dose, *d* for single dose, and *α*/*β* assumed as 10 for the tumour [14,15]:

$$EQD_{2}=\frac{d+\alpha ∕\beta }{2+\alpha ∕\beta }$$

Chemotherapy was also administered according to local practices, which means either concomitantly to radiotherapy as recommended by prospective randomized control trials (RCTs) [3,16,17,18,19] and international guidelines [20,21] or in the sequential mode. Usually, patients received immunotherapy if programmed death ligand 1 (PD-L1) expression was 1% or higher. In some cases, however, in accordance with the PACIFIC study [4,5], the local tumour board decided to administer immunotherapy even if the PD-L1 status was negative. In accordance with the above stated aim of this analysis, the cohort was stratified by the administration of ICIs.

### 2.3. Endpoints and Statistics

The clinical endpoints of this investigation were OS, progression-free survival (PFS), locoregional control (LRC) [22], and treatment-related toxicity. OS was calculated until death or the last follow-up visit. PFS was defined as the time until the first tumour relapse at any site. LRC was the period until the occurrence of an event within the radiation. Pulmonary and oesophageal side effects were the adverse events of special interest (AESI). The first term includes pneumonitis of any cause as well as fibrosis, interstitial lung disease, and pneumonia. The second is a general term for dysphagia, oesophagitis, and fibrotic stricture. For data analysis, graph plotting and hypothesis testing using the programming language R (version 4.3.1; R Foundation for Statistical Computing, Vienna, Austria) including the open source libraries “tidyverse”, “survival”, and “survminer” were used. OS, PFS, and LRC rates were estimated with the Kaplan–Meier method from the date of pathological diagnosis. Subgroups were fitted with the Cox proportional hazard regression model. The hazard ratio (HR) including the two-sided 95% confidence intervals together with the *p*-value of the log-rank test are reported. Wilcoxon–Mann–Whitney Test for ordinal and categorial variables (IBM SPSS version 29.0) was performed to test the statistically significant differences of baseline and treatment characteristics. A *p*-value below the 5% level was considered significant.

## 3. Results

### 3.1. Patients

**Figure 1:**
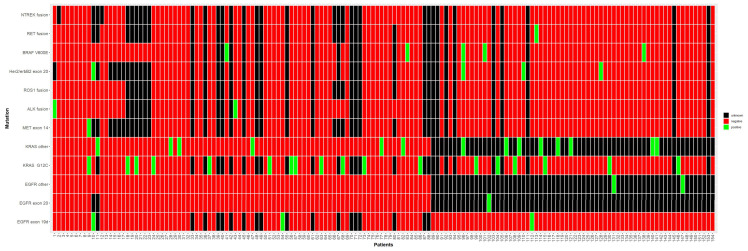
Mutational status of the most common driver mutations. In 154/188 (82%) patients, data on at least one driver mutation were available (colour code: green = positive, red = negative, black = unknown). While 18/32 (56%) patients were mutated in KRAS G12C, 14/32 (44%) had other KRAS mutations (G12A, G12D, G12S, G12V, Q61H) including 2/32 (6%) unspecified mutations. One patient was simultaneously mutated in METexon14. A second patient had co-mutations in her2/erbB2exon20 plus and BRAF V600E.

Between 2020/03 and 2023/04, 12/14 (86%) Austrian radiation-oncology centres recruited 243 patients in a nationwide registry for LA-NSCLC. Eight clinics provided information on the patients‘ mutational status of the most common druggable targets including EGFR, KRAS, MET, ALK, ROS1, Her2, BRAF V600E, RET, and NTRK [8]. This corresponds to 154/188 (82%) patients from the whole ALLSTAR cohort (Figure 1). Among these, 32/154 (21%) had KRAS mutations. While 18/32 (56%) patients had a G12C mutation, 12/32 (38%) patients had different alterations such as G12A, G12D, G12S, G12V, and Gln61His, and 2/32 (6%) were unspecified. One patient had a tumour that was simultaneously mutated in METexon14. A second patient also had mutations in her2/erbB2exon20 and BRAF V600E (Supplementary Table S1). None of the KRASmt patients had a co-mutation for EGFR or ALK/ROS1 (Figure 1). All patients had at least one follow-up visit three months after finishing CR(I)T as required by the protocol so that the median follow-up (FUP) amounted to 25.9 months (range: 3.3–46.4). The mean number of patients per centre was 4 (range: 1–14), with two centres contributing 25/32 (78%) of the patients. The number of females in both groups was on a par with males and the median age was 64 and 62 years, respectively. Most patients had a good performance status (ECOG 0–1), except for one patient with ECOG 2 in the non-immunotherapy group. Except for 1/32 (3%) patient in the non-immunotherapy group, all patients had adenocarcinomas. The PD-L1 status was available in more than 90% of the patients. Tumour size and nodal status as well as UICC stages were similarly distributed in both groups (Table 1).

### 3.2. Radiochemotherapy

Although concomitant chemoradiotherapy (cCRT) is the standard of care, the majority of patients in this cohort received CRT in the sequential mode (63% and 60%, respectively). As for systemic therapy, carboplatinum or cisplatinum was combined with either pemetrexed, taxane, or vinorelbine (Table 2). The median number of cycles was 2 (range: 1–4) and 3 (range: 2–6) in immunotherapy and non-immunotherapy group, respectively. No significant differences could be found between groups (supplementary Table S2). While RT was administered using volumetric arc therapy (VMAT) or IMRT in most cases, only 1/32 (3%) patient in the immunotherapy group had 3D-RT. Altogether, 12/32 (38%) of the patients received high-dose radiation > 66 Gy. The GTV_Tumour_ did not differ significantly between groups (*p*-value = 0.448), whereas the total median EQD2_Tumour_ was significantly higher in the immunotherapy group (66 Gy versus 48 Gy; *p*-value = 0.008). Similarly, total median EQD2_Lymphnodes_ was significantly different between groups (57 Gy versus 45 Gy; *p*-value = 0.027; Table 2).

### 3.3. Immunotherapy

Twenty-seven of the thirty-two patients were treated with immunotherapy. The PD-L1 status was known in 30/32 (94%) patients. Durvalumab was administered in 23/27 (85%) of the cases, whereas Pembrolizumab was used in the other 4/27 (15%) patients. One patient who was also PD-L1 positive was not treated with ICI because of a reduced general condition with permanent oxygen supply. The median latency between the end of RT and the initiation of Durvalumab was 14 days (range: 1–57). The 23 Durvalumab patients received a median of 6 cycles (range: 1–27). Five of the twenty-three patients (22%) had received between 21 and 27 cycles of Durvalumab and were, therefore, regarded as having finished therapy. For patient convenience, practices have changed towards extending the treatment interval to four weeks with a fixed dose of 1500 mg so that, in some cases, the cycle number tested in PACIFIC was not reached. Similar to a previous publication by the ALLSTAR group [10], information on treatment interruptions were calculated using the administration of cortisone as a proxy. The most reliable data were available on the 23/27 (85%) patients who received Durvalumab. A total of 5 of the 23 (22%) patients received cortisone for one of the following reasons: pneumonitis (3), COPD exacerbation (1), unknown (1). In three of the four patients who were treated with Pembrolizumab, immunotherapy was started before RT as the patients were initially deemed operable and, therefore, treated according to Keynote-671 [23]. The remaining patient started Pembrolizumab 46 days after the end of RT. The median cycle number for Pembrolizumab was 16 (range: 8–40).

### 3.4. OS, PFS, LRC, and Toxicity

**Figure 2:**
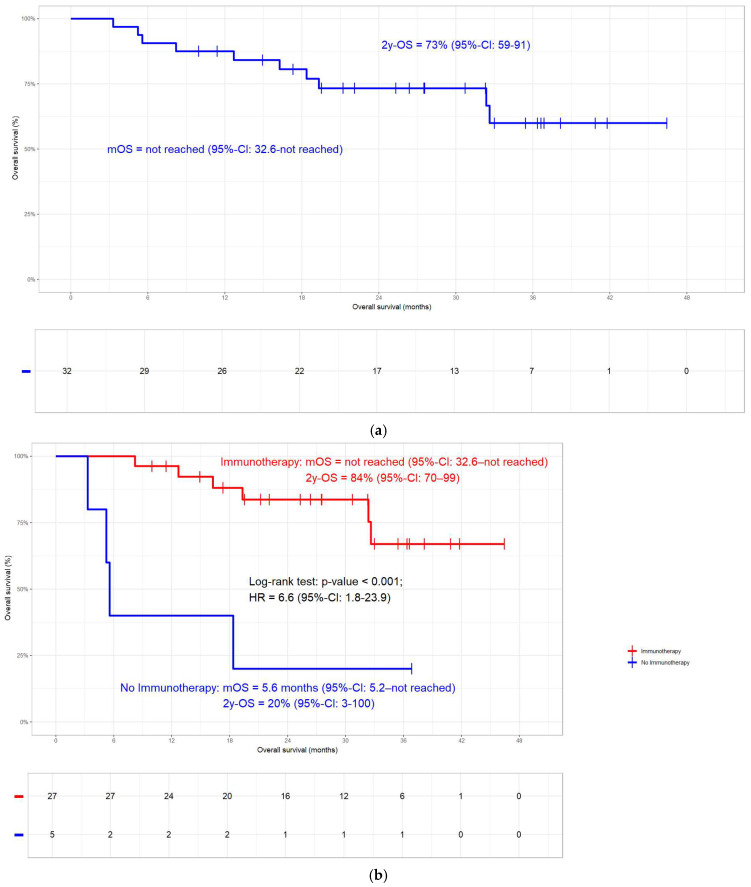
(**a**) Overall survival (OS). In the whole cohort (N = 32), the 2-year OS rate was 73%. (**b**) Overall survival. At 84%, the 2-year OS rate in KRASmt patients who received immunotherapy was significantly better compared to the 20% in those without ICI (N = 32; log-rank test; *p* < 0.001).

**Figure 3:**
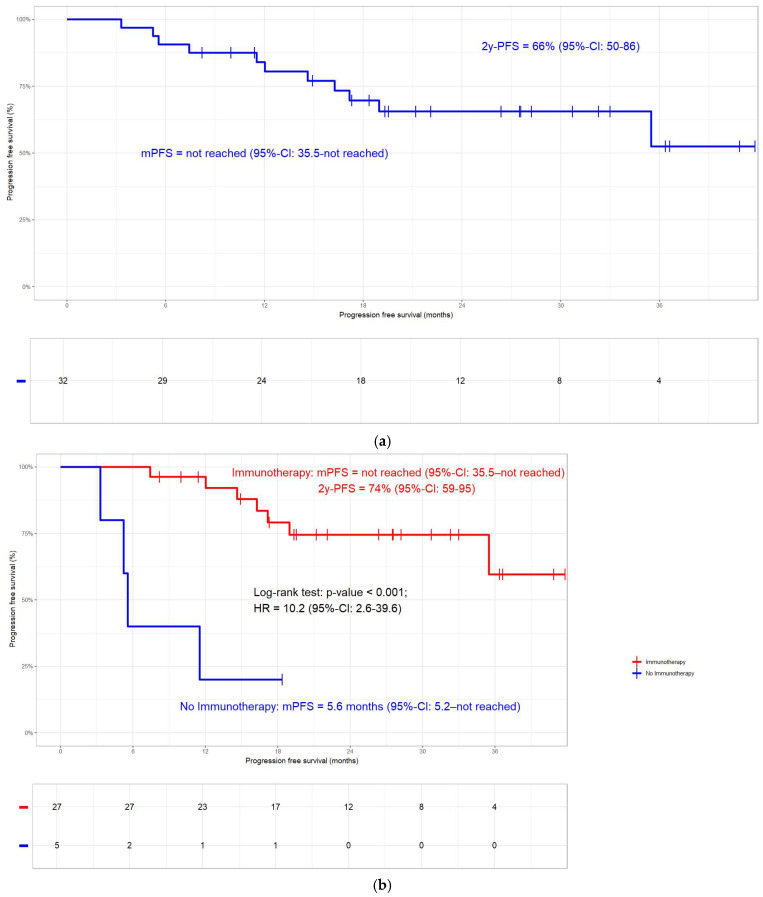
(**a**) Progression-free survival (PFS). In the whole cohort (N = 32), the 2-year PFS rate was 66%. (**b**) Progression-free survival. At 74%, the 2-year PFS rate in KRASmt patients who received immunotherapy was significantly better compared to the 20% in those without (N = 32; log-rank test; *p* < 0.001).

**Figure 4:**
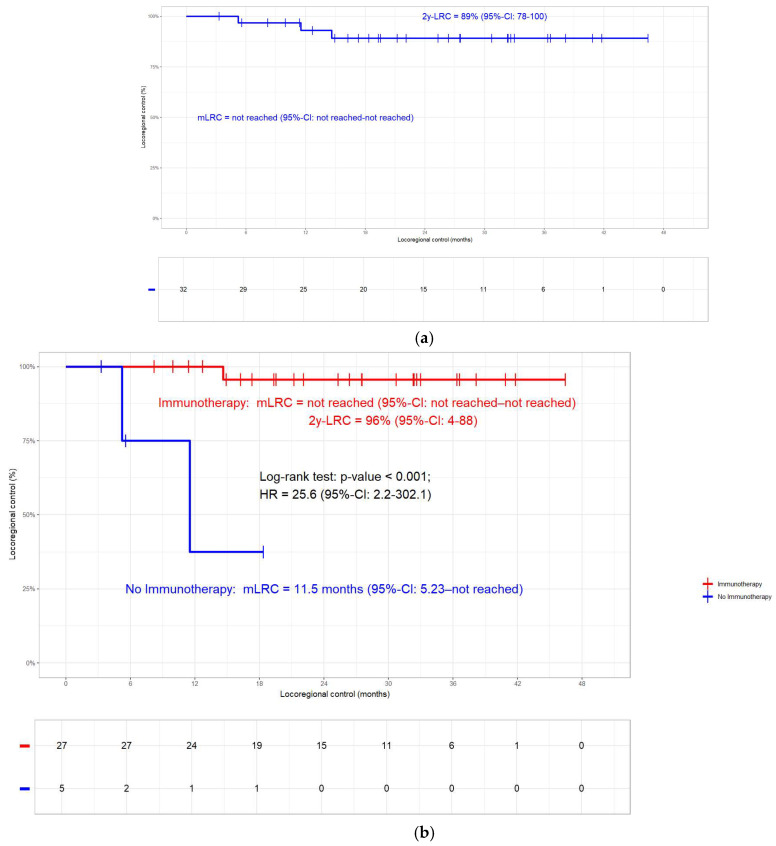
(**a**) Locoregional control (LRC). In the whole cohort (N = 32), the 2-year LRC rate was 89%. (**b**) Loco-regional control. At 96%, the 2-year LRC rate in KRASmt patients who received immunotherapy was significantly better compared to the 38% in those without (N = 32; log-rank test; *p* < 0.001).

In general, the 2-year OS rate was 73% (mOS not reached, Figure 2a). In the non-immunotherapy group (N = 5), 4 deaths occurred compared to the 6 events in the 27 patients with ICI. At 84%, the 2-year OS rate of patients who received ICI was significantly better compared to the 20% in those without (N = 32; log-rank test; *p* < 0.001; Figure 2b). In the whole cohort, the 2-year PFS rate was 66% (Figure 3a). Patients without ICI (N = 5; 4 progressions) had more progressions compared to those with ICI (N = 27; 6 progressions). At 75%, the 2-year PFS rate in patients who received ICI was significantly better compared to the 20% in those without ICI (N = 32; log-rank test; *p* < 0.001; Figure 3b). Overall, the 2-year LRC rate was 89% (Figure 4a). In accordance with the other clinical endpoints, immunotherapy also improved the 2-year LRC rate significantly (96% versus 38%; N = 32; log-rank test; *p* < 0.001; Figure 4b). Of the 12/32 patients (38%) who had received radiation doses > 66 Gy, none had a locoregional relapse. In the other 20 patients, 5 (25%) events occurred (log-rank *p*-value = 0.116; Supplementary Figure S1). Similar to a previous publication by our group [10], oesophageal and pulmonary toxicity were the adverse events of special interest, with immunotherapy patients having a slightly higher percentage of side effects (Table 3).

### 3.5. KRAS G12C

With 18/32 (56%) patients, KRAS G12C was the most common mutation in our cohort. The comparison between these patients and the ones with other genetic alterations revealed no difference with respect to OS (log-rank *p*-value = 0.222; Supplementary Figure S2), PFS (log-rank *p*-value = 0.231; Supplementary Figure S3, and LRC (log-rank *p*-value = 0.442; Supplementary Figure S4).

## 4. Discussion

The current study demonstrates that patients with KRAS mutations benefit from ICI in terms of OS, PFS, and LRC (Figure 2b, Figure 3b and Figure 4b). In line with a previous publication by our group [10], LRC can additionally be improved by a moderate dose escalation with the total radiation doses > 66 Gy (supplementary Figure S1). No difference in clinical outcome could be detected between patients with KRAS G12C mutation and other types (Supplementary Figures S2–S4).

Our analysis is one of the very few that was conducted in UICC stage III patients who were treated with immunotherapy [7,24,25], so that comparisons with larger series, which mainly come from the pre-immunotherapy era and consist of UICC IV patients, are only partially meaningful [26,27]. According to a recent review on druggable targets, the percentage of any KRAS mutation in a Western population is about 25% [8]. With 32 patients, the current cohort is one of the largest series in KRASmt patients presenting with unresectable stage III NSCLC published in the era of immunotherapy thus far [7,24,25]. In line with Barsouk et al. [24], patients with EGFR or ALK/ROS1 mutations were excluded from the current analysis as they have poorer prognosis and do not respond well to immunotherapy [28]. KRAS mutations are usually associated with the female sex, adenocarcinoma [7,24,27,29,30], and smoking [30]; therefore, patients with these clinical features are overrepresented compared to the average NSCLC stage III population [4,10,11]. This is also true for the current cohort with 97% adenocarcinomas, which is slightly higher than reported by Guo [7]. Women make up 50% of the current cohort, which is less than comparable studies in KRASmt patients [24,25] but higher than in common NSCLC populations. A meta-analysis of more than 7000 patients states that PD-L1 expression is higher in KRASmt than in KRASwt patients [31], which is also corroborated by our data with 25/32 (78%) PD-L1-positive patients. Therefore, some authors hypothesized that KRASmt may modify response to ICI [29], which could explain the favourable outcome in KRASmt patients with immunotherapy [9,29]. In contrast, a very recent review including NSCLC stage IV patients mainly [32] found that PD-L1 had no impact on OS and PFS in KRASmt patients.

In our cohort, the PD-L1 status was known in 94% of the patients, which is—with two exceptions [24,29]—substantially higher than in most studies with comparable cohort sizes [7,9,25]. It is also much higher than the PD-L1 diagnosis rate in prospective studies [6,33] and RWD analyses [13,34,35,36,37]. With a 14-day median latency between the end of CRT and the initiation of Durvalumab, the gap is the same as in the overall ALLSTAR cohort [10] and a Japanese RWD [38]. This is about half the delay reported in other KRAS studies [7,24]. Furthermore, compared to RWD studies published in the wake of the PACIFIC trial [4,5] with an average of around 40 days [11,13,34,39,40,41] up to 72 days in the Spanish EAP study [36], the lag-time in ALLSTAR KRASmt seems very short. Twenty-two percent of the patients were regarded as having finished ICI therapy after 21 to 27 cycles of Durvalumab. A bi-weekly administration of Durvalumab—as initially tested by PACIFIC [4,5]—would require 26 cycles without interruptions. Over the years, treatment practices have changed by extending the interval to four weeks with a fixed dose of 1500 mg per cycle. This may—at least partly—explain, why patients with less than 26 cycles may be regarded as having finished ICI treatment. In our cohort, 22% of the patients terminated immunotherapy, which is a rate that is comparatively lower than in other KRAS populations [7,24]. Durvalumab treatment interruption, which has a negative impact on outcome [42], happened in 22% of our patients. Therapy was finally stopped due to AESIs in half of these patients. This is a similar rate as in PACIFIC [4,5] and another KRAS cohort [7]. Although lying in the range of the published literature [6,12,36,38,40,41,43,44], our results have to be regarded with caution, since they are an estimate by proxy.

The median follow-up period of 25.9 months for the whole cohort is well in line with published reports on KRASmt patients [7,24] and RWD studies on Durvalumab in general [36,38]. As opposed to most studies in this field, the index date of the time to event analyses was the day of pathological diagnosis and not the start of Durvalumab as in [7,24], which may make OS, PFS, and LRC rates in our cohort seem longer than in published studies. The 84% 2-year OS rate (mOS not reached, Figure 2b) for patients treated with ICI is on the upper edge of the published literature [11,12,34,35,37,39,40,45]. With 75% PFS at 2 years, patients who received immunotherapy had significantly better outcome than those without (Figure 3b). Half of the patients with extrathoracic metastases had brain metastases, which corroborates previously published data demonstrating a higher susceptibility for intracranial relapse in KRASmt patients [30]. Some reports state that KRAS mutation is a negative prognosticator for PFS [30]. Consistently, the 6.3 months mPFS for KRASmt patients without ICI in one report [24] is almost the same as the 5.6 months in our cohort. Barsouk et al. concluded that the higher rate of disease progressions between CRT and the start of ICI might be the reason for this finding [24]. This very same study also reports that if KRASmt patients receive ICI, they have a similar PFS as KRASwt patients [24]. This is in line with a meta-analysis including 13,000 patients with mainly advanced NSCLC and a small proportion of—depending on the study—7.6% to 17.5% stage III [32]. In contrast, Cortiula et al. show in their report on patients with NSCLC stage III only that immunotherapy did not have an impact on PFS [25]. At 89%, the 2-year LRC rate was relatively high in the current subcohort (Figure 4a), in which the administration of immunotherapy resulted in a highly significant difference (Figure 4b). While this is in line with reports on two other data sets [38,39], LRC was not impacted by ICI in the whole ALLSTAR population [10]. Unfortunately, in the current context, this is only a speculation since our registry lacks data on both the exact percentage of PD-L1 expression and the TP53 status. Moreover, none of the patients treated with total radiation doses > 66 Gy had a relapse (Supplementary Figure S1), which—probably due to the small patient numbers—was not significant but showed a strong trend. With a 66 Gy median total dose in the immunotherapy group, our cohort was on the upper dose limit compared to PACIFIC [4] and other studies [7,24,26,30]. In 38% of the patients, high-dose radiation beyond 66 Gy EQD2 was applied. The proportion in this subcohort is in the same range as in the whole cohort [10] and substantially higher than the approximately 10% in comparable RWD studies [11,34,40,44,46]. Hypofractionated accelerated radiation dose escalation, in general, may shorten overall treatment time and enhance LRC rates. At the same time, however, it harbours the potential of supra-additive pulmonary toxicity when administered together with immunotherapy [47,48].

As the current analysis is based on a nationwide registry, its strength is that it demonstrates the efficacy of ICI treatment in a representative KRASmt patient cohort. With 32 patients, this subcohort is one of the largest in the field. Patient data were collected prospectively, which contrasts other RWD studies. Although baseline characteristics did not differ significantly between groups, the small patient number in the non-immunotherapy group may constitute a bias that may have had an impact on our results. Additionally, the same restrictions as described elsewhere [10] for the whole ALLSTAR patient population also apply to this subcohort. Firstly, as three of the participating centres recruited 85% of the patients, which is also reflected in this subcohort, our results may only partially represent the therapeutic panorama for KRASmt patients in Austria. Secondly, for lack of manpower, a measurement bias may be introduced by the fact that patients in clinical registries are not followed up as meticulously as in prospective trials, which may result in seemingly higher OS, PFS, and LRC rates in comparison to the literature.

Our findings together with other reports [7,24,25] are elucidating in the context of tailored therapies. Since KRAS G12C is the most frequent oncogenic driver mutation [27], Sotorasib and Adagrasib, which are currently approved in NSCLC UICC stage IV [8], combined with ICI could also potentially become future therapeutic options in stage III.

## 5. Conclusions

KRASmt patients could potentially benefit from ICI in terms of PFS and OS so that it seems reasonable to offer this treatment similar to individuals without actionable genetic drivers. Additionally, total radiation dose escalation may improve LRC. Larger prospective multicentre studies with a focus on this subset of patients are warranted.

## Figures and Tables

**Table 1:** Patients. Adenocarcinoma (AC), squamous cell carcinoma (SCC), not otherwise specified (NOS).

Patients N = 32.				
		Immunotherapy	No Immunotherapy	*p*-Value
		N = 27 (%)	N = 5 (%)	
Sex	male	14 (52)	2 (40)	0.687
	female	13 (48)	3 (60)	
Age (years)	median	64	62	0.614
	range	52–74	57–70	
Smoking status	never	1 (4)	0 (0)	0.389
	ex	18 (67)	5 (100)	
	current	8 (29)	0 (0)	
ECOG	0–1	27 (100)	4 (80)	0.077
	2–3	0 (0)	1 (20)	
Histology	AC	27 (100)	4 (80)	0.511
	SCC	0 (0)	0 (0)	
	NOS	0 (0)	1 (20)	
PD-L1	<1%	2 (8)	3 (60)	0.220
	>1%	24 (89)	1 (20)	
	unknown	1 (4)	1 (20)	
T-stage	Tis	0 (0)	0 (0)	0.220
	1	6 (22)	0 (0)	
	2	3 (11)	0 (0)	
	3	8 (30)	2 (40)	
	4	10 (37)	3 (60)	
N-stage	0	3 (11)	0 (0)	0.920
	1	0 (0)	1 (20)	
	2	19 (70)	3 (60)	
	3	5 (19)	1 (20)	
M-stage	0	27 (100)	5 (100)	n.a.
	1	0 (0)	0 (0)	
UICC	IIIa	9 (33)	1 (20)	0.687
	IIIb	13 (48)	3 (60)	
	IIIc	5 (19)	1 (20)	

**Table 2:** Treatment. Immune checkpoint inhibition (ICI), radiotherapy (RT), biologically equivalent dose in 2 Gy fractions (EQD2), gross tumour volume (GTV), chemoradiotherapy (CRT), volumetric arc therapy (VMAT), intensity modulated radiotherapy (IMRT).

Treatment N = 32					
			Immunotherapy	No Immunotherapy	*p*-Value
			N = 27 (%)	N = 5 (%)	
Treatment sequence	concomitant CRT		10 (37)	2 (40)	0.920
	sequential CRT		17 (63)	3 (60)	
ICI	Durvalumab		23 (85)	0 (0)	n.a.
	Pembrolizumab		4 (15)	0 (0)	
RT Technique	VMAT/IMRT		26 (96)	5 (100)	0.920
	3-D		1 (4)	0 (0)	
Tumour	EQD2 (Gy)	median	66	48	0.008
		range	45–100	25–67	
	GTV (mL)	median	45	70	0.448
		range	1–484	1–130	
Lymph nodes	EQD2 (Gy)	median	57	45	0.027
		range	33–66	24–60	
	GTV (mL)	median	25	83	0.078
		range	1–408	20–473	
Elective nodal irradiation	EQD2 (Gy)	median	41	53	n.a.
		range	33–53	none	
	GTV (mL)	median	137	137	n.a.
		range	26–176	none	

**Table 3:** Toxicity.

Toxicity N = 32				
		Immunotherapy	No Immunotherapy	*p*-Value
		N = 27 (%)	N = 5 (%)	
Esophagitis	Grade 1	4 (11)	0 (0)	0.614
	Grade 2	7 (26)	3 (60)	
	Grade 3	1 (4)	0 (0)	
	Grade 4	0 (0)	0 (0)	
	Grade 5	0 (0)	0 (0)	
Pneumonitis	Grade 1	3 (11)	1 (20)	0.960
	Grade 2	1 (4)	0 (0)	
	Grade 3	0 (0)	0 (0)	
	Grade 4	0 (0)	0 (0)	
	Grade 5	0 (0)	0 (0)	
Hematologic	any grade	1 (4)	0 (0)	n.a.
Other	any grade	2 (8)	0 (0)	n.a.

## Data Availability

The original contributions presented in this study are included in the article/Supplementary Materials. Further inquiries can be directed to the corresponding author(s).

## Supplementary Materials

The following supporting information can be downloaded at: https://www.mdpi.com/article/10.3390/jcm14030945/s1, Figure S1: Locoregional control: KRAS mutated patients who received total doses > 66 Gy had better locoregional control than those with 66 Gy or less (N = 32; log-rank test *p*-value = 0.116); Figure S2: KRAS G12C versus other mutations: overall survival was not significantly different; Figure S3: KRAS G12C versus other mutations: progression free survival was not significantly different; Figure S4: KRAS G12C versus other mutations: locoregional control was not significantly different; Table S1: KRASmt and co-mutations: One patients was simultaneously mutated in MET Exon14. A second patient had a double co-mutation in her2erbB2exon20 and BRAF 600E. As for KRASmt patient, 18/32 (54%) had G12C mutation and 15/32 (46%) had different alterations: G12A, G12D, G12S, G12V, Q61H. In two patients the KRAS mutation was not further specified; Table S2: Chemotherapy.

## Abbreviations

AESI	adverse events of special interest
ALLSTAR	Austrian radio-oncological lung cancer study association registry
CR(I)T	chemoradioimmunotherapy
CRT	chemoradiotherapy
cCRT	concomitant chemoradiotherapy
EAP	early access programme
eCRF	electronic case report form
EQD2	biologically equivalent dose in 2 Gy fractions
FUP	follow-up
ICI	immune checkpoint inhibition
IMRT	intensity modulated radiotherapy
KRAS	Kirsten rat sarcoma virus
KRASmt	KRAS mutation
KRASwt	KRAS wild-type
LA-NSCLC	locally advanced non-small cell lung cancer
LRC	locoregional control
mOS	median overall survival
NSCLC	non-small cell lung cancer
PD-L1	programmed death ligand 1
PFT	pulmonary function tests
RCT	randomized control trial
RT	radiotherapy
SoC	standard of care
VMAT	volumetric arc therapy
WBDC	web-based data capture system

## References

[1] Sung H., Ferlay J., Siegel R.L., Laversanne M., Soerjomataram I., Jemal A., Bray F. (2021). Global Cancer Statistics 2020: GLOBOCAN Estimates of Incidence and Mortality Worldwide for 36 Cancers in 185 Countries. CA Cancer J. Clin..

[2] Bradley J.D., Paulus R., Komaki R., Masters G., Blumenschein G., Schild S., Bogart J., Hu C., Forster K., Magliocco A. (2015). Standard-dose versus high-dose conformal radiotherapy with concurrent and consolidation carboplatin plus paclitaxel with or without cetuximab for patients with stage IIIA or IIIB non-small-cell lung cancer (RTOG 0617): A randomised, two-by-two factorial phase 3 study. Lancet Oncol..

[3] Auperin A., Le Pechoux C., Rolland E., Curran W.J., Furuse K., Fournel P., Belderbos J., Clamon G., Ulutin H.C., Paulus R. (2010). Meta-analysis of concomitant versus sequential radiochemotherapy in locally advanced non-small-cell lung cancer. J. Clin. Oncol..

[4] Antonia S.J., Villegas A., Daniel D., Vicente D., Murakami S., Hui R., Yokoi T., Chiappori A., Lee K.H., de Wit M. (2017). Durvalumab after Chemoradiotherapy in Stage III Non-Small-Cell Lung Cancer. N. Engl. J. Med..

[5] Antonia S.J., Villegas A., Daniel D., Vicente D., Murakami S., Hui R., Kurata T., Chiappori A., Lee K.H., de Wit M. (2018). Overall Survival with Durvalumab after Chemoradiotherapy in Stage III NSCLC. N. Engl. J. Med..

[6] Spigel D.R., Faivre-Finn C., Gray J.E., Vicente D., Planchard D., Paz-Ares L., Vansteenkiste J.F., Garassino M.C., Hui R., Quantin X. (2022). Five-Year Survival Outcomes From the PACIFIC Trial: Durvalumab After Chemoradiotherapy in Stage III Non-Small-Cell Lung Cancer. J. Clin. Oncol..

[7] Guo M.Z., Murray J.C., Ghanem P., Voong K.R., Hales R.K., Ettinger D., Lam V.K., Hann C.L., Forde P.M., Brahmer J.R. (2022). Definitive Chemoradiation and Durvalumab Consolidation for Locally Advanced, Unresectable-mutated Non-Small Cell Lung Cancer. Clin. Lung Cancer.

[8] Tan A.C., Tan D.S.W. (2022). Targeted Therapies for Lung Cancer Patients With Oncogenic Driver Molecular Alterations. J. Clin. Oncol..

[9] Jeanson A., Tomasini P., Souquet-Bressand M., Brandone N., Boucekine M., Grangeon M., Chaleat S., Khobta N., Milia J., Mhanna L. (2019). Efficacy of Immune Checkpoint Inhibitors in KRAS-Mutant Non-Small Cell Lung Cancer (NSCLC). J. Thorac. Oncol..

[10] Zehentmayr F., Feurstein P., Ruznic E., Langer B., Grambozov B., Klebermass M., Hüpfel H., Feichtinger J., Minasch D., Heilmann M. (2024). Durvalumab impacts progression-free survival while high-dose radiation > 66 Gy improves local control without excess toxicity in unresectable NSCLC stage III: Real-world data from the A ustrian radio-oncologica l l ung cancer st udy a ssociation r egistry (ALLSTAR). Radiother. Oncol..

[11] Girard N., Bar J., Garrido P., Garassino M.C., McDonald F., Mornex F., Filippi A.R., Smit H.J.M., Peters S., Field J.K. (2023). Treatment Characteristics and Real-World Progression-Free Survival in Patients With Unresectable Stage III NSCLC Who Received Durvalumab After Chemoradiotherapy: Findings From the PACIFIC-R Study. J. Thorac. Oncol..

[12] Park C.K., Oh H.J., Kim Y.C., Kim Y.H., Ahn S.J., Jeong W.G., Lee J.Y., Lee J.C., Choi C.M., Ji W.J. (2023). Korean Real-World Data on Patients With Unresectable Stage III NSCLC Treated With Durvalumab After Chemoradiotherapy: PACIFIC-KR. J. Thorac. Oncol..

[13] Preti B.T.B., Sanatani M.S., Breadner D., Lakkunarajah S., Scott C., Esmonde-White C., Mcarthur E., Rodrigues G., Chaudhary M., Mutsaers A. (2023). Real-World Analysis of Durvalumab after Chemoradiation in Stage III Non-Small-Cell Lung Cancer. Curr. Oncol..

[14] Bentzen S.M., Dorr W., Gahbauer R., Howell R.W., Joiner M.C., Jones B., Jones D.T., van der Kogel A.J., Wambersie A., Whitmore G. (2012). Bioeffect modeling and equieffective dose concepts in radiation oncology–terminology, quantities and units. Radiother. Oncol..

[15] Fowler J.F., Tome W.A., Fenwick J.D., Mehta M.P. (2004). A challenge to traditional radiation oncology. Int. J. Radiat. Oncol. Biol. Phys..

[16] Furuse K., Fukuoka M., Kawahara M., Nishikawa H., Takada Y., Kudoh S., Katagami N., Ariyoshi Y. (1999). Phase III study of concurrent versus sequential thoracic radiotherapy in combination with mitomycin, vindesine, and cisplatin in unresectable stage III non-small-cell lung cancer. J. Clin. Oncol..

[17] Fournel P., Robinet G., Thomas P., Souquet P.J., Lena H., Vergnenegre A., Delhoume J.Y., Le Treut J., Silvani J.A., Dansin E. (2005). Randomized phase III trial of sequential chemoradiotherapy compared with concurrent chemoradiotherapy in locally advanced non-small-cell lung cancer: Groupe Lyon-Saint-Etienne d’Oncologie Thoracique-Groupe Francais de Pneumo-Cancerologie NPC 95-01 Study. J. Clin. Oncol..

[18] Zatloukal P., Petruzelka L., Zemanova M., Havel L., Janku F., Judas L., Kubik A., Krepela E., Fiala P., Pecen L. (2004). Concurrent versus sequential chemoradiotherapy with cisplatin and vinorelbine in locally advanced non-small cell lung cancer: A randomized study. Lung Cancer.

[19] Curran W.J. (2011). Sequential vs Concurrent Chemoradiation for Stage III Non-Small Cell Lung Cancer: Randomized Phase III Trial RTOG 9410. J. Natl. Cancer Inst..

[20] Daly M.E., Singh N., Ismaila N., Antonoff M.B., Arenberg D.A., Bradley J., David E., Detterbeck F., Früh M., Gubens M.A. (2022). Management of Stage III Non-Small-Cell Lung Cancer: ASCO Guideline. J. Clin. Oncol..

[21] Remon J., Soria J.C., Peters S., Comm E.G. (2021). Early and locally advanced non-small-cell lung cancer: An update of the ESMO Clinical Practice Guidelines focusing on diagnosis, staging, systemic and local therapy. Ann. Oncol..

[22] Machtay M., Paulus R., Moughan J., Komaki R., Bradley J.E., Choy H., Albain K., Movsas B., Sause W.T., Curran W.J. (2012). Defining local-regional control and its importance in locally advanced non-small cell lung carcinoma. J. Thorac. Oncol..

[23] Wakelee H., Liberman M., Kato T., Tsuboi M., Lee S.H., Gao S., Chen K.N., Dooms C., Majem M., Eigendorff E. (2023). Perioperative Pembrolizumab for Early-Stage Non-Small-Cell Lung Cancer. N. Engl. J. Med..

[24] Barsouk A., Friedes C., Iocolano M., Doucette A., Cohen R.B., Robinson K.W., D’Avella C.A., Marmarelis M.E., Kosteva J.A., Singh A.P. (2024). Plunging Into the PACIFIC: Outcomes of Patients with Unresectable KRAS-Mutated Non-Small Cell Lung Cancer Following Definitive Chemoradiation and Durvalumab Consolidation. Clin. Lung Cancer.

[25] Cortiula F., De Ruysscher D., Steens M., Wijsman R., van der Wekken A., Alberti M., Hendriks L.E.L. (2023). Adjuvant durvalumab after concurrent chemoradiotherapy for patients with unresectable stage III NSCLC harbouring uncommon genomic alterations. Eur. J. Cancer.

[26] Hallqvist A., Enlund F., Andersson C., Sjogren H., Hussein A., Holmberg E., Nyman J. (2012). Mutated KRAS Is an Independent Negative Prognostic Factor for Survival in NSCLC Stage III Disease Treated with High-Dose Radiotherapy. Lung Cancer Int..

[27] Isaksson J., Berglund A., Louie K., Willén L., Hamidian A., Edsj A., Enlund F., Planck M., Vikström A., Johansson M. (2023). KRAS G12C Mutant Non-Small Cell Lung Cancer Linked to Female Sex and High Risk of CNS Metastasis: Population-based Demographics and Survival Data From the National Swedish Lung Cancer Registry. Clin. Lung Cancer.

[28] Remon J., Hendriks L.E.L. (2021). Targeted therapies for unresectable stage III non-small cell lung cancer. Mediastinum.

[29] Cronin-Fenton D., Dalvi T., Movva N., Pedersen L., Hansen H., Fryzek J., Hedgeman E., Mellemgaard A., Rasmussen T.R., Shire N. (2021). PD-L1 expression, and mutations and survival among stage III unresected non-small cell lung cancer patients: A Danish cohort study. Sci. Rep..

[30] Yagishita S., Horinouchi H., Sunami K.S., Kanda S., Fujiwara Y., Nokihara H., Yamamoto N., Sumi M., Shiraishi K., Kohno T. (2015). Impact of mutation on response and outcome of patients with stageIII non-squamous non-small cell lung cancer. Cancer Sci..

[31] Lan B., Ma C., Zhang C., Chai S., Wang P., Ding L., Wang K. (2018). Association between PD-L1 expression and driver gene status in non-small-cell lung cancer: A meta-analysis. Oncotarget.

[32] Zhao R., Shu Y., Xu W., Jiang F.X., Ran P.C., Pan L.Y., Wang J.L., Wang W.H., Zhao J., Wang Y.H. (2024). The efficacy of immunotherapy in non-small cell lung cancer with KRAS mutation: A systematic review and meta-analysis. Cancer Cell Int..

[33] Garassino M.C., Mazieres J., Reck M., Chouaid C., Bischoff H., Reinmuth N., Cove-Smith L., Mansy T., Cortinovis D., Migliorino M.R. (2022). Durvalumab After Sequential Chemoradiotherapy in Stage III, Unresectable NSCLC: The Phase 2 PACIFIC-6 Trial. J. Thorac. Oncol..

[34] Filippi A.R., Bar J., Chouaid C., Christoph D.C., Field J.K., Fietkau R., Garassino M.C., Garrido P., Haakensen V.D., Kao S. (2024). Real-world outcomes with durvalumab after chemoradiotherapy in patients with unresectable stage III NSCLC: Interim analysis of overall survival from PACIFIC-R. ESMO Open.

[35] Borghetti P., Volpi G., Facheris G., Cossali G., Mataj E., La Mattina S., Singh N., Imbrescia J., Bonù M.L., Tomasini D. (2023). Unresectable stage III non-small cell lung cancer: Could durvalumab be safe and effective in real-life clinical scenarios? Results of a single-center experience. Front. Oncol..

[36] Rueda A.G., Taus A., Alvarez R.A., Bernabé-Caro R., Chara L., López-Brea M., Vilà L., González M.A.S., Aldagalán A.D.D., Herrera B.E. (2024). The S-REAL study: Spanish real-world data on unresectable stage III NSCLC patients treated with durvalumab after chemoradiotherapy. Clin. Transl. Oncol..

[37] Faehling M., Schumann C., Christopoulos P., Hoffknecht P., Alt J., Horn M., Eisenmann S., Schlenska-Lange A., Schütt P., Steger F. (2020). Durvalumab after definitive chemoradiotherapy in locally advanced unresectable non-small cell lung cancer (NSCLC): Real-world data on survival and safety from the German expanded-access program (EAP). Lung Cancer.

[38] Abe T., Saito S., Iino M., Aoshika T., Ryuno Y., Ohta T., Igari M., Hirai R., Kumazaki Y., Miura Y. (2021). Effect of durvalumab on local control after concurrent chemoradiotherapy for locally advanced non-small cell lung cancer in comparison with chemoradiotherapy alone. Thorac. Cancer.

[39] Huang Y.Q., Zhao J.J., Soon Y.Y., Wong A., Aminkeng F., Ang Y., Asokumaran Y., Low J.L., Lee M., Choo J.R.E. (2022). Real-world experience of consolidation durvalumab after concurrent chemoradiotherapy in stage III non-small cell lung cancer. Thorac. Cancer.

[40] Waterhouse D., Yong C.D.C., Frankart A., Brannman L., Mulrooney T., Robert N., Aguilar K.M., Ndukum J., Cotarla I. (2023). Durvalumab real-world treatment patterns and outcomes in patients with stage III non-small-cell lung cancer treated in a US community setting. Future Oncol..

[41] Offin M., Shaverdian N., Rimner A., Lobaugh S., Shepherd A.F., Simone C.B., Gelblum D.Y., Wu A.J., Lee N., Kris M.G. (2020). Clinical outcomes, local-regional control and the role for metastasis-directed therapies in stage III non-small cell lung cancers treated with chemoradiation and durvalumab. Radiother. Oncol..

[42] Xu T., Wu L.R., Gandhi S., Jing W., Nguyen Q.N., Chen A.L., Chang J.Y., Nurieva R., Sheshadri A., Altan M. (2022). Treatment-related pulmonary adverse events induced by chemoradiation and Durvalumab affect survival in locally advanced non-small cell lung cancer. Radiother. Oncol..

[43] Garassino M.C., Mazieres J., Reck M., Chouaid C., Bischoff H., Reinmuth N., Cove-Smith L.S., Mansy T., Cortinovis D.L., Migliorino M.R. (2023). Durvalumab (durva) after sequential chemoradiotherapy (CRT) in patients (pts) with unresectable stage III NSCLC: Final analysis from PACIFIC-6. Ann. Oncol..

[44] Bruni A., Scotti V., Borghetti P., Vagge S., Cozzi S., D’Angelo E., Levra N.G., Fozza A., Taraborrelli M., Piperno G. (2021). A Real-World, Multicenter, Observational Retrospective Study of Durvalumab After Concomitant or Sequential Chemoradiation for Unresectable Stage III Non-Small Cell Lung Cancer. Front. Oncol..

[45] Mooradian M., Allen A., Cai L., Xiao Y., Chander P. (2022). Real-world outcomes with durvalumab (durva) after chemoradiotherapy (CRT) in patients with unresectable stage III NSCLC (SPOTLIGHT). Ann. Oncol..

[46] Jung H.A., Noh J.M., Sun J.M., Lee S.H., Ahn J.S., Ahn M.J., Pyo H., Ahn Y.C., Park K. (2020). Real world data of durvalumab consolidation after chemoradiotherapy in stage III non-small-cell lung cancer. Lung Cancer.

[47] Wass R., Hochmair M., Kaiser B., Grambozov B., Feurstein P., Weiss G., Moosbrugger R., Sedlmayer F., Lamprecht B., Studnicka M. (2022). Durvalumab after Sequential High Dose Chemoradiotherapy versus Standard of Care (SoC) for Stage III NSCLC: A Bi-Centric Trospective Comparison Focusing on Pulmonary Toxicity. Cancers.

[48] Landman Y., Jacobi O., Kurman N., Yariv O., Peretz I., Rotem O., Dudnik E., Zer A., Allen A.M. (2021). Durvalumab after concurrent chemotherapy and high-dose radiotherapy for locally advanced non-small cell lung cancer. Oncoimmunology.

