# Supplementary material for: Immunotherapy Improves Clinical Outcome in Kirsten Rat Sarcoma Virus-Mutated Patients with Unresectable Non-Small Cell Lung Cancer Stage III: A Subcohort Analysis of the Austrian Radio-Oncological Lung Cancer Study Association Registry (ALLSTAR)
Source: J Clin Med. 2025 Feb 1;14(3):945. doi: 10.3390/jcm14030945 (PMC11818499; doi:10.3390/jcm14030945)
Supplement: Supplementary file 1 [file jcm-14-00945-s001.zip › 03 supplementary tables final.pdf]

Supplementary Table 1. KRASmt and co-mutations. One patient was simultaneously mutated in METexon14. A second patient had a double co-mutation in her2/erbB2exon20 plus and BRAF V600E. As for the KRASmt patients, 18/32 (54%) had a G12C mutation and 15/32 (46%) had different alterations: G12A, G12D, G12S, G12V, Q61H. In two patients, the KRAS mutation was not further specified.

**Co-mutations**		
Gene mutation	n	%
EGFR exon 19del	0	0
EGFR exon 20	0	0
EGFR other	0	0
KRAS G12C	18	56
KRAS other	14	44
MET exon 14	1	3
ALK fusion	0	0
ROS1 fusion	0	0
Her2/erbB2 exon 20	1	3
BRAF V600E	1	3
RET fusion	0	0
NTRK fusion	0	0

Supplementary Table 2.

**Chemotherapy N = 32**				
**Chemotherapy prior to RT**		**Immunotherapy** N = 17	**No Immunotherapy** N = 3	**p-value**
Substances	carboplatinum/pemetrexed	16****	1	0.763
	carboplatinum/taxane	0	0	
	carboplatinum/gemcitabine	0	0	
	carboplatinum/vinorelbine	0	0	
	cisplatinum/pemetrexed	0	2	
	cisplatinum/taxane	1***	0	
	cisplatinum/gemcitabine	0	0	
	cisplatinum/vinorelbine	0	0	
	vinorelbine mono	0	0	
	pemetrexed mono	0	0	
Cycles	median	2	3	0.511
	range	1 - 4	2 - 6	
**Chemotherapy simultaneous to RT**		N = 10	N = 2	**p-value**
Substances	carboplatinum/pemetrexed	5*§	0	0.725
	carboplatinum/taxane	0	0	
	carboplatinum/gemcitabine	0	0	
	carboplatinum/vinorelbine	0	1	
	cisplatinum/pemetrexed	5**	0	
	cisplatinum/taxane	0	0	
	cisplatinum/gemcitabine	0	0	
	cisplatinum/vinorelbine	0	1	
	vinorelbine mono	0	0	
	pemetrexed mono	0	0	
Cycles	median	2	3	0.725
	range	1 - 4	2 - 4	

*2 patients received 2 cycles before RT
**1 patient received 1 cycle before RT
***this patient also received 2 cycles after RT
**** 2 patients also received 2 cycles after RT
§1 patient received 1 cycle after RT
