# Supplementary material for: Immunotherapy Improves Clinical Outcome in Kirsten Rat Sarcoma Virus-Mutated Patients with Unresectable Non-Small Cell Lung Cancer Stage III: A Subcohort Analysis of the Austrian Radio-Oncological Lung Cancer Study Association Registry (ALLSTAR)
Source: J Clin Med. 2025 Feb 1;14(3):945. doi: 10.3390/jcm14030945 (PMC11818499; doi:10.3390/jcm14030945)

Supplementary Figure 1. Loco-regional control: KRAS-mutated patients who received total radiation doses >66 Gy had better locoregional control than those with 66 Gy or less (N = 32; log-rank test; p-value = 0.116).

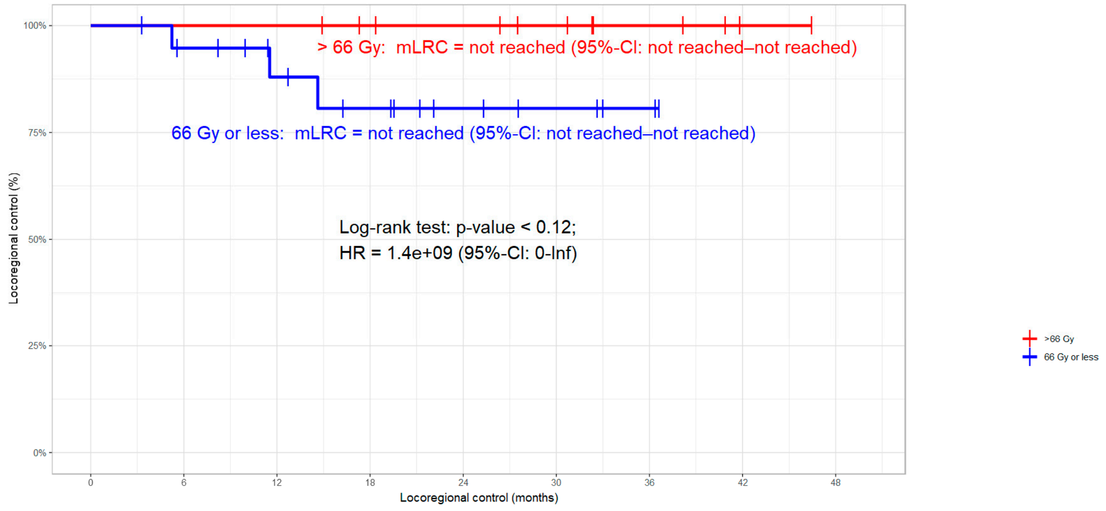

red	12	12	12	10	9	7	4	1	0
blue	20	17	13	10	6	4	2	0	0

Supplementary Figure 2. KRAS G12C versus other mutations: overall survival was not significantly different.

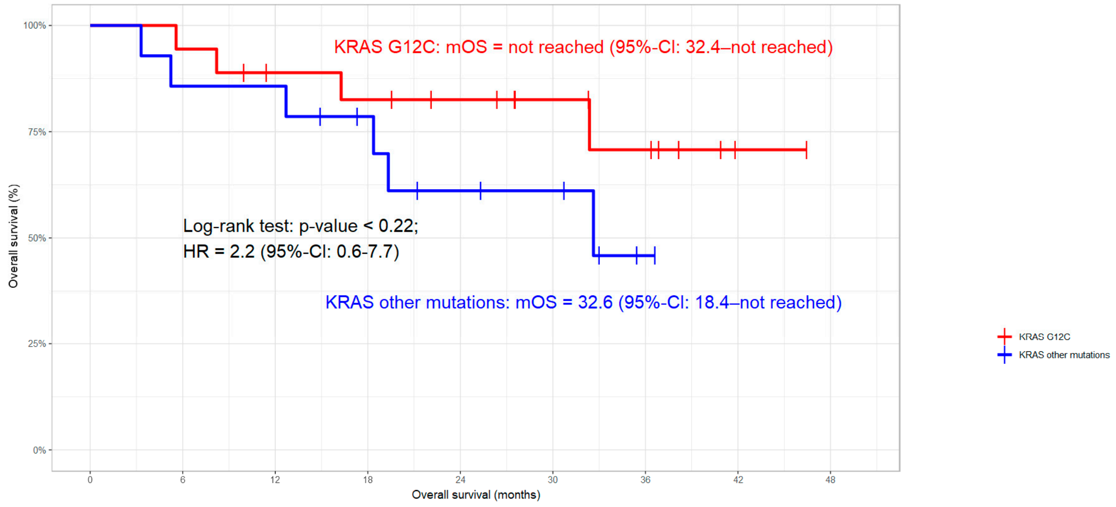

red	18	17	14	13	11	8	6	1	0
blue	14	12	12	9	6	5	1	0	0

Supplementary Figure 3. KRAS G12C versus other mutations: progression-free survival was not significantly different.

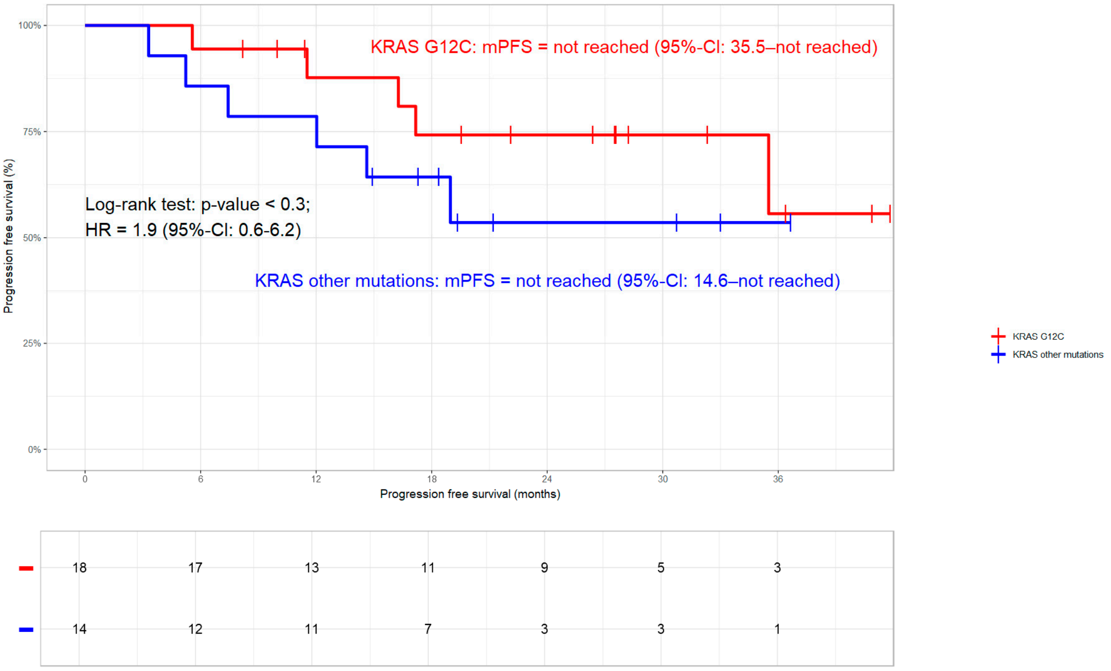

Supplementary Figure 4. KRAS G12C versus other mutations: loco-regional control was not significantly different.

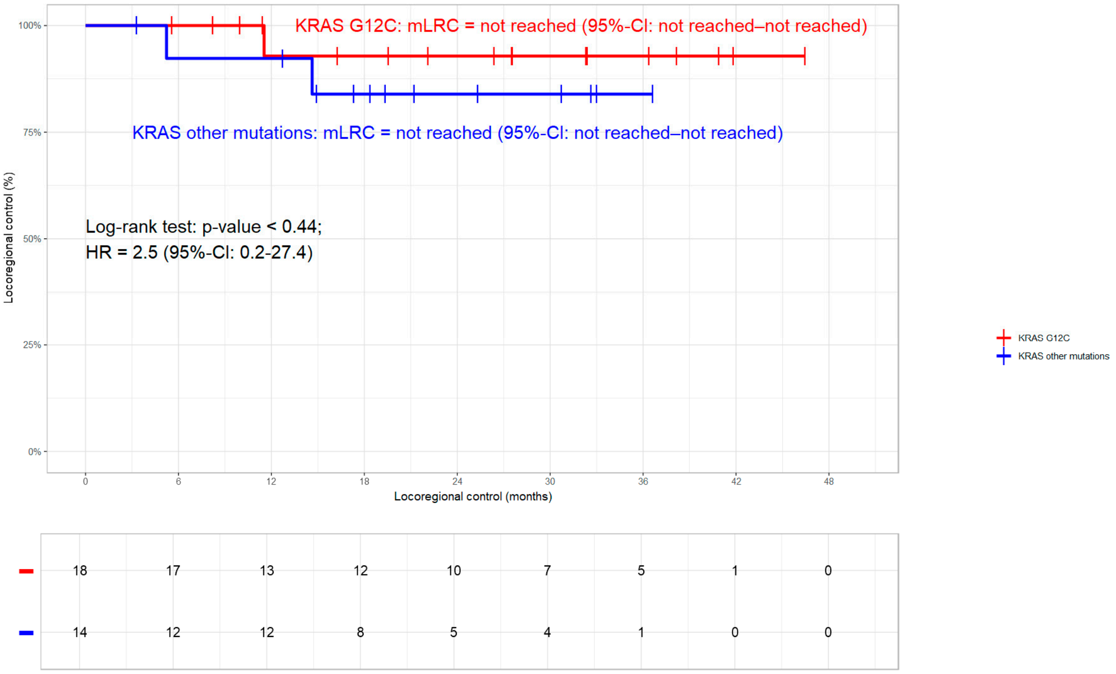

Supplement: Supplementary file 1 [file jcm-14-00945-s001.zip › 04 supplementary figures final.pdf]
